# Risk-based management of international sporting events during the COVID-19 pandemic

**DOI:** 10.2471/BLT.23.290034

**Published:** 2024-07-04

**Authors:** Albis Francesco Gabrielli, Amaia Artazcoz Glaria, Maria Borodina, Lucia Mullen, Crystal R Watson, Amanda Kobokovich, Ninglan Wang

**Affiliations:** a Border Health and Mass Gatherings, Country Readiness and Strenghtening, World Health Organization Health Emergencies Programme, World Health Organization, Avenue Appia 20, 1211 Geneva 27, Switzerland.; b The Johns Hopkins Center for Health Security, Johns Hopkins Bloomberg School of Public Health, Baltimore, United States of America.

## Abstract

Mass gatherings include a diverse range of events such as sporting competitions, religious ceremonies, entertainment activities, political rallies and cultural celebrations, which have important implications for population well-being. However, if not managed properly, these events can amplify health risks including those related to communicable diseases, and place undue strain on health systems in host countries and potentially in attendees’ home countries, upon their return. The coronavirus disease 2019 (COVID-19) pandemic has provided a unique opportunity to evaluate the risk factors associated with mass gatherings and the effectiveness of applying mitigation measures during infectious disease emergencies. The pandemic has also allowed event organizers and health officials to identify best practices for mass gathering planning in host countries. To guide decisions about whether to hold, postpone, modify or cancel a mass gathering during the COVID-19 pandemic, the World Health Organization and its partners developed normative guidance and derivative tools promoting a risk-based approach to mass gathering planning. This approach involves three steps to guide decision-making around mass gatherings: risk evaluation, risk mitigation and risk communication. The approach was applied in the planning and execution of several mass gathering events, including the Tokyo 2020 and Beijing 2022 Olympic and Paralympic Games. Lessons identified from these large-scale international events offer insights into the planning and implementation of mass gathering events during a pandemic, and the broader impacts of such events on society. These lessons may also further inform and refine planning for future mass gatherings.

## Résumé

Les rassemblements de masse désignent un large éventail d'événements tels que des compétitions sportives, cérémonies religieuses, activités de divertissement, manifestations politiques et fêtes culturelles. Tous ont un impact considérable sur le bien-être de la population. Toutefois, s'ils ne sont pas gérés correctement, ils peuvent augmenter les risques sanitaires, notamment concernant les maladies transmissibles, et exercer une pression excessive sur les systèmes de santé des pays hôtes, voire sur ceux des pays d'origine des participants après leur retour. La pandémie de maladie à coronavirus 2019 (COVID-19) a offert une occasion unique d'évaluer les facteurs de risque associés aux rassemblements de masse, ainsi que l'efficacité des mesures visant à limiter la propagation dans des situations d'urgence liées à des maladies infectieuses. Cette pandémie a également permis aux organisateurs d'événements et responsables de santé d'identifier les bonnes pratiques à appliquer dans les pays hôtes pendant les rassemblements de masse. Afin de guider les décisions relatives au maintien, au report, à la modification ou à l'annulation d'un rassemblement de masse durant la pandémie de COVID-19, l'Organisation mondiale de la Santé et ses partenaires ont mis au point des orientations normatives et des outils dérivés favorisant une approche tenant compte des risques au moment de la planification. Cette approche comprend trois étapes contribuant à la prise de décision: l'évaluation, la réduction et la communication des risques. Elle a été déployée lors de la planification et de l'exécution de nombreux rassemblements de masse, comme les Jeux olympiques et paralympiques de Tokyo 2020 et Beijing 2022. Les leçons tirées de ces événements internationaux à grande échelle fournissent des informations sur leur organisation et leur mise en œuvre en cas de pandémie, ainsi que les impacts de tels événements sur la société. Elles sont en outre susceptibles de faciliter et d'améliorer la planification des futurs rassemblements de masse.

## Resumen

Las concentraciones masivas incluyen una gran variedad de eventos, como competiciones deportivas, ceremonias religiosas, actividades de entretenimiento, mítines políticos y celebraciones culturales, que tienen importantes implicaciones para el bienestar de la población. Sin embargo, si no se gestionan adecuadamente, estos eventos pueden amplificar los riesgos para la salud, incluidos los relacionados con las enfermedades transmisibles, y suponer una carga excesiva para los sistemas sanitarios de los países anfitriones y, potencialmente, de los países de origen de los participantes a su regreso. La pandemia de la enfermedad por coronavirus de 2019 (COVID-19) ha brindado una oportunidad única para evaluar los factores de riesgo asociados a las concentraciones masivas y la eficacia de aplicar medidas de mitigación durante las emergencias por enfermedades infecciosas. La pandemia también ha permitido a los organizadores de eventos y a las autoridades sanitarias identificar las mejores prácticas para la planificación de concentraciones masivas en los países anfitriones. Para orientar las decisiones sobre la celebración, el aplazamiento, la modificación o la cancelación de una concentración masiva durante la pandemia de la COVID-19, la Organización Mundial de la Salud y sus asociados elaboraron orientaciones normativas y herramientas derivadas que promueven un enfoque de la planificación de concentraciones masivas basado en los riesgos. Este enfoque consta de tres pasos para orientar la toma de decisiones en torno a las concentraciones masivas: la evaluación, la mitigación y la comunicación de riesgos. El enfoque se aplicó en la planificación y ejecución de varias concentraciones masivas, incluidos los Juegos Olímpicos y Paralímpicos de Tokio 2020 y Pekín 2022. Las conclusiones extraídas de estos eventos internacionales a gran escala permiten comprender mejor la planificación y ejecución de concentraciones masivas durante una pandemia, así como las repercusiones más generales de estos eventos en la sociedad. Estas lecciones también pueden informar y perfeccionar la planificación de futuras concentraciones masivas.

## ملخص

تشمل التجمعات الغفيرة مجموعة متنوعة من الأحداث مثل المسابقات الرياضية، والاحتفالات الدينية، والأنشطة الترفيهية، والمسيرات السياسية، والاحتفالات الثقافية، وهي ذات انعكاسات مهمة على رفاهية السكان. ومع ذلك، فإنه إذا لم تتم إدارة هذه الأحداث بشكل صحيح، فإنها يمكن أن تؤدي إلى تضاعف المخاطر الصحية بما في ذلك تلك المتعلقة بالأمراض المعدية، وتضع ضغوطًا بلا حدود على النظم الصحية في الدول المضيفة، ومن المحتمل كذلك في دول الحاضرين الأصلية، عند عودتهم لها. أتاحت جائحة مرض فيروس كورونا 2019 (كوفيد 19) فرصة فريدة لتقييم عوامل الخطر المرتبطة بالتجمعات الغفيرة، وفعالية تطبيق تدابير التخفيف أثناء حالات الطوارئ الخاصة بالأمراض المعدية. كما سمح الوباء لمنظمي الأحداث ومسؤولي الصحة بالتعرف على أفضل الممارسات للتخطيط التجمعات الغفيرة في الدول المضيفة. لتوجيه القرارات المتعلقة بعقد تجمع غفير، أو تأجيله، أو تعديله، أو إلغائه أثناء جائحة كوفيد 19، قامت منظمة الصحة العالمية (WHO) وشركاؤها بوضع توجيهات معيارية، وأدوات مشتقة تعزز الأسلوب القائم على المخاطر في التخطيط للتجمعات الغفيرة. يتضمن هذا الأسلوب ثلاث خطوات لتوجيه عملية صنع القرار بخصوص التجمعات الغفيرة، ألا وهي: تقييم المخاطر، وتخفيف المخاطر، والإبلاغ عن المخاطر. وتم تطبيق هذا الأسلوب أثناء تخطيط العديد من أحداث التجمعات الغفيرة وتنفيذها، بما في ذلك دورتي الألعاب الأولمبية والبارالمبية في طوكيو 2020 وبكين 2022. إن الدروس المستفادة من هذه الأحداث الدولية واسعة النطاق تقدم نظرات متعمقة على تخطيط أحداث التجمعات الغفيرة وتنفيذها أثناء الجائحة، والانعكاسات الأوسع لمثل هذه الأحداث على المجتمع. كما أن هذه الدروس قد تؤدي أيضًا إلى التوعية والتحسين المستقبلي بشكل أكبر للتخطيط للتجمعات الغفيرة.

## 摘要

大型集会包括对民众福祉具有重要影响的体育比赛、宗教仪式、娱乐活动、政治集会和文化庆典等各种活动。然而，如果管理不当，这些活动可能会增加卫生风险（包括与传染病有关的风险），并对东道国的卫生系统造成过大压力，甚至可能会在参与者回国后对其本国造成过大压力。2019 冠状病毒病 (COVID-19) 大流行为我们提供了评估与大型集会相关的风险因素以及在传染病突发情况下采取缓解措施的有效性的独特机会。大流行还有助于活动组织者和卫生官员找出在东道国策划大型集会的最佳做法。为指导在 COVID-19 大流行期间决定是否举行、推迟、变更或取消大型集会，世界卫生组织及其合作伙伴制定了规范性指南和衍生工具，从而提出了策划大型集会的风险管理方法。这一方法涉及就大型集会进行决策指导的三个步骤：风险评估、风险缓解和风险沟通。该方法已被应用于多项大型集会活动的策划和执行过程中，包括 2020 年东京奥运会和 2022 年北京冬季奥运会和残奥会。从这些大型国际活动中吸取的经验教训为大流行期间策划和实施大型集会活动以及了解此类活动会对社会造成的更广泛影响提供了见解。这些经验教训还可为未来完善大型集会的策划提供更多的依据。

## Резюме

В число массовых мероприятий входит целый ряд событий, таких как спортивные соревнования, религиозные церемонии, развлекательные мероприятия, политические митинги и культурные праздники, которые оказывают важное влияние на благосостояние населения. Однако при отсутствии надлежащего управления эти мероприятия могут усилить риски для здоровья, в том числе связанные с инфекционными заболеваниями, а также создать чрезмерную нагрузку на системы здравоохранения в принимающих странах и, возможно, в странах проживания участников после их возвращения. Пандемия коронавирусной инфекции в 2019 году (COVID-19) предоставила уникальную возможность оценить факторы риска, связанные с массовыми мероприятиями, и эффективность применения мер по снижению риска во время чрезвычайных ситуаций, связанных с инфекционными заболеваниями. Пандемия также позволила организаторам мероприятий и сотрудникам органов здравоохранения определить оптимальные методы планирования массовых мероприятий в принимающих странах. Для принятия решений о проведении, переносе, изменении или отмене массовых мероприятий во время пандемии COVID-19 Всемирная организация здравоохранения и ее партнеры разработали нормативное руководство и производные инструменты, способствующие применению подхода к планированию массовых мероприятий на основе рисков. Этот подход предполагает три этапа принятия решений о массовых мероприятиях: оценку рисков, снижение рисков и информирование о рисках. Подход применялся при планировании и проведении нескольких массовых мероприятий, включая Олимпийские и Паралимпийские игры 2020 года в Токио и 2022 года в Пекине. Уроки, извлеченные из этих крупномасштабных международных событий, позволяют понять, как планировать и проводить массовые мероприятия во время пандемии и как они влияют на общество в целом. Эти выводы также могут послужить основой для планирования массовых мероприятий в будущем.

## Introduction

Mass gatherings are characterized by the concentration of people at a specific location for a specific purpose over a set period. Such gatherings encompass a diverse range of events, including sporting competitions, religious ceremonies, entertainment events, political rallies and cultural celebrations. Mass gatherings are not merely recreational; rather, they have important implications for population health and well-being. If not managed properly, however, mass gatherings may amplify health risks including those related to communicable diseases, and placing undue strain on health systems in host countries and potentially in attendees’ home countries, upon their return. The coronavirus disease 2019 (COVID-19) pandemic and previous large-scale infectious disease emergencies, such as severe acute respiratory syndrome (SARS) (2003), Middle East respiratory syndrome (MERS) (2012), H1N1 influenza (2009) and Zika (2016), have underscored how epidemics and pandemics can disrupt mass gatherings, and how such events could potentially worsen the spread and impact of high-risk outbreaks.^1^^,^^2^ As such, the World Health Organization (WHO) has prioritized identifying risk-based approaches to hold mass gatherings safely during pandemics and other public health emergencies of international concern, and published relevant guidance.^3^

The COVID-19 pandemic has provided a unique opportunity to evaluate risk factors associated with mass gatherings and the effectiveness of risk mitigation measures applied to such events during major outbreaks. The pandemic has also enabled event organizers and health experts to identify best practices for mass gathering planning in host countries and highlighted the positive long-term benefits, or legacies,^3^ of mass gathering events when they are implemented thoughtfully, with appropriate planning, resources and engagement.

To support decisions about whether to hold, postpone, modify or cancel a mass gathering during COVID-19, WHO has adapted existing recommendations on mass gatherings to the pandemic context and developed normative guidance and related tools. The guidance promotes risk-based approaches to mass gathering planning amid an evolving public health emergency.

## Planning mass gatherings

In line with WHO’s general guidance,^3^ when planning mass gatherings, decision-makers should identify public health risks and assess them in relation to how risks may affect and be amplified by these events, to devise adequate precautionary measures. In the context of COVID-19, this exercise focused on the likelihood and consequences of SARS coronavirus 2 (SARS-CoV-2) spreading in the host country and participants’ home countries. Through implementing precautionary measures, WHO aimed at making mass gatherings as safe as possible by preventing surges in COVID-19 cases and deaths. 

Every mass gathering is unique; as such, no one-size-fits-all solution to ensuring safety exists, which is particularly true for risks associated with communicable diseases. Therefore, in the face of COVID-19’s dynamic evolution, the global health community expected that precautionary measures would vary between countries and over time. Nevertheless, having consolidated, consistent and structured approaches to manage risk associated with planned events is essential both in terms of preparedness and response.^4^ Thus such approaches should be adaptable and flexible, to swiftly guide the decision-making process, allow for systematic analysis of COVID-19 risks, weigh potential trade-offs associated with risk mitigation, and enable safe delivery of events across diverse contexts and epidemiological conditions.^5^^–^^11^

With these considerations in mind, in early 2020 WHO convened global scientists (from academia, public health agencies and research institutions, among others) as part of the COVID-19 Mass Gatherings expert group to adapt existing general risk-based guidance on mass gatherings, and conceptualize a tailored approach that would support decision-making around mass gatherings in the context of the COVID-19 pandemic.^12^ WHO initiated these efforts in response to requests from Member States, international organizations and event planners. Although WHO does not have a mandate to enforce any action with respect to holding, delaying or modifying mass gathering events, the Organization does play a role in developing technical guidance on evidence-based best practices and disseminating it among relevant stakeholders to support informed decision-making.^13^ WHO’s role in this regard aligns with Article 43 of the International Health Regulations (2005), which makes provisions for States Parties to implement measures concerning travellers taking part in mass congregations.^14^

## A risk-based approach to COVID-19

As a derivative product of WHO’s general guidance on mass gatherings, the Organization adopted the COVID-19 risk-based approach as an official policy and published it as interim guidance; the principles of the approach were also articulated in a WHO policy brief.^10^^,^^11^ The approach is adaptable to gatherings of any size and type – as well as to any SARS-CoV-2 transmission scenario – and consists of three steps. First, risk evaluation, which aims at identifying and quantifying the baseline risks associated with the gathering. Second, risk mitigation, which proposes a series of precautionary measures aimed at decreasing the baseline risk of the gathering. Third, risk communication, which prompts the timely and proactive dissemination of information on the process, rationale, purpose and limits of the precautionary measures adopted, with the aim of enhancing adherence by event attendees.

The risk evaluation step involves examining key characteristics of the mass gathering event in question, as well as the context in which the event takes place. To assess the likelihood of SARS-CoV-2 transmission and its public health consequences, this step encourages decision-makers to consider key aspects such as the epidemiological context of the host community; influxes of mass gathering participants and spectators; characteristics of the venue; health needs and risk profiles of attendees; and potential risk-taking behaviours connected to the event. Other critical considerations include local health system capacities; vaccination coverage; variants of concern; and levels of compliance with public health and social measures in place in the host country.

Risk mitigation focuses on a package of multisectoral actions that aim at enhancing public health and preserving safety; these may be implemented before, during and after the event. Examples of relevant mitigation measures include establishing or strengthening coordination between all event stakeholders, bolstering capacities among designated staff and establishing event-based surveillance mechanisms. Risk mitigation also embodies the value of enforcing basic measures such as physical distancing, hand hygiene, mask use, screening attendees at port of entry or at venues, and monitoring attendee movement during the event.

The risk communication step offers guidance on deploying robust community engagement strategies before, during and after the event in question, such as using visual displays or audio reminders, website postings, social media or other communication platforms. Risk communication may also entail social media monitoring and infodemic management. Notably, this step underscores the importance of conveying to attendees and event planners the need for personal responsibility and respectful behaviour, as well as the message that eliminating all potential risk is impossible.

To further operationalize the guidance, upon WHO’s request and under its technical oversight, the WHO Collaborating Centre for Global Health Security at the Johns Hopkins University, with support from the WHO COVID-19 Mass Gatherings expert group, developed several risk assessment tools that were published as WHO products and underwent subsequent revisions. In addition to the COVID-19 Mass Gathering Risk Assessment tool for generic events, these actors developed two specific Risk Assessment Tools tailored for sporting and religious events.^15^^–^^17^ These tools account for the unique planning considerations associated with such gatherings. Sporting events, for example, may present varying levels of risk for athletes, team officials and spectators due to the nature of the sport in question.^18^^,^^19^ Physical distancing may also be impossible for certain athletes during competition. Additionally, while athletes are typically young, healthy individuals, spectators may include more medically vulnerable individuals. 

As the pandemic unfolded, the same authors systematically updated both the guidance and the tools to reflect the evolving nature of WHO recommendations on COVID-19. Best practices on planning and implementation of mass gatherings emerged as events were implemented and reviewed, and efforts were made to improve the usability of the tools.

Through different normative mechanisms, WHO has recommended that Member States adopt the risk-based approach when planning for mass gatherings during the COVID-19 pandemic. To monitor mass gathering decision-making and implementation through COVID-19, WHO and the WHO Collaborating Centre for Global Health Security at the Johns Hopkins University developed and maintained a global database of mass gatherings since the beginning of the pandemic. By 31 December 2023, when the database was discontinued a few months after WHO declared that COVID-19 no longer constituted a public health emergency of international concern (5 May 2023), 164 WHO Member States and 11 other countries, territories and areas reported having applied the risk-based approach to at least one mass gathering event to decide whether or how it should be held. By the same date, the number of recorded mass gatherings for 2020–2023 was 5852, of which 52.3% (3060) had applied WHO’s risk-based approach in their decision-making process.^20^^,^^21^ Notably, the database mainly captured larger events with international participation; as such, the above figure cannot be considered representative of the volume of mass gatherings held globally during the pandemic.

## Tokyo 2020 and Beijing 2022

Among the mass gatherings whose planning and execution relied on WHO’s risk-based approach, two major international sporting events held during the COVID-19 pandemic merit particular consideration due to their size, international participation and high visibility: the 2020 Summer Olympic and Paralympic Games held in Tokyo, Japan in July−September 2021, and the 2022 Winter Olympic and Paralympic Games held in Beijing, China in February−March 2022. Close, collaborative partnerships between the International Olympic Committee, the International Paralympic Committee, the countries hosting these events and WHO formed the cornerstone of these efforts.

In preparation for Tokyo 2020 and Beijing 2022, through the International Olympic Committee and the International Paralympic Committee, the two host countries’ organizing committees invited WHO to join dedicated working groups. These working groups convened all actors involved in the events, and offered a platform for policy dialogue related to health in general and COVID-19 in particular. WHO’s role involved sharing guidance and providing tailored advice to the other actors, notably to the national organizing committees that represented each country’s government – and that are ultimately responsible for the events. These efforts enabled informed decision-making for health-related aspects of both events, and facilitated the development of event-specific normative products that were based on WHO recommendations. Notably, the International Olympic Committee and the International Paralympic Committee, in collaboration with the organizing committees of Tokyo 2020 and Beijing 2022, published two sets of Playbooks^22^^,^^23^ featuring protocols for public safety, COVID-19 vaccination, routine testing, laboratory sequencing, onsite precautionary measures and in-country travel restrictions. The Playbooks also introduced innovative mitigation measures such as bubbles and closed loops (that is, approaches that allow close, in-person interactions only among a defined group of people, thus limiting the risk of transmission from and to people external to the bubble or loop), and articulated roles and responsibilities for COVID-19 compliance officers.

In line with its mandate, WHO also activated an internal coordination mechanism to assess and monitor health-related risks, and established daily reporting systems between its headquarters, regional offices and relevant WHO country offices, based on which WHO produced daily and weekly situational reports. Through its convening power, WHO facilitated close cooperation among relevant international, national and local health agencies and other stakeholders for both events. WHO also scaled up event-based surveillance for COVID-19, other outbreak-prone communicable diseases and other acute events,^24^ with the goals of detecting and assessing emergent signals of public health concern, monitoring infodemics, and quantifying and analysing relevant public health information from digital and social media (such as Twitter – now X – Facebook, Instagram, YouTube, blogs and other forums). These methods of event-based surveillance enabled event organizers to track the spread of misinformation about COVID-19 and address concerns about public safety. Additionally, the robust risk communication measures implemented by the host countries’ organizing committees in conjunction with both events and through different channels (visual displays, audio reminders, website postings, mobile applications, social media and helplines among others) enabled securing buy-in and cooperation from spectators, athletes, event staff, the media and other stakeholders. Constant communication and information sharing between Member States, event organizers, WHO and the public were essential components of implementing the risk-based approach, as this approach facilitated taking evidence-based decisions.

## Challenges and lessons learnt

During and after Tokyo 2020 and Beijing 2022, WHO informally convened at regular intervals the WHO COVID-19 Mass Gatherings expert group to share advice, review iterations of technical guidance and contribute to discussions among all stakeholders. Notably, sharing information with partners about potential public health risks, documenting best practices, reviewing challenges and identifying lessons further cemented the long-term benefits generated by both events, that is, their legacy.^3^ The main reflections of these focus group discussions coordinated by WHO are presented below.

Overall, Tokyo 2020 and Beijing 2022 yielded rich lessons and best practices for planning complex international events amid an evolving public health emergency, notably with regard to risk-based decision-making. These lessons, in turn, may be more broadly applicable to future mass gatherings of all types.

First, the decision to modify, postpone or cancel a mass gathering often depends on financial considerations in addition to health and safety concerns. Though applying the risk-based approach can help facilitate safe and successful event delivery – which, in turn, may generate revenue for host countries and organizers and yield important psychosocial benefits for participants and spectators – applying precautionary measures clearly also requires significant levels of human and financial resources. Event organizers, therefore, must account for these factors when planning gatherings of all sizes.

Second, planning considerations should not be limited to the event itself; rather, they must also encompass the social context in which the event takes place. For example, although the organizers of Tokyo 2020 and Beijing 2022 successfully enforced strict safety protocols within event venues, promoting community adherence to such protocols outside official venues proved challenging. Planners must therefore apply a holistic approach, paying close attention to public health and social measures; transportation between events; accommodations; and individual behaviours that might lead to side gatherings, unplanned congregation and unstructured socialization in public spaces or close quarters.

Third, event organizers should ensure a transparent planning environment, open to all relevant stakeholders (athletes, spectators, organizers, etc.) and sectors (health, security, sport, business, transportation, etc.). All these stakeholders should be involved in event planning from its inception to ensure that all considerations and concerns are identified early, shared and incorporated into decision-making processes around the gathering in question. In many cases, earning stakeholder buy-in also encourages acceptance of changes to safety policies and plans – which, in turn, may improve compliance with precautionary measures at the event itself.

Fourth, communication is a particularly important component of delivering safe and successful mass gatherings. This aspect includes disseminating public health messages regularly, demonstrating transparency in the decision-making process, highlighting the importance of complying with risk mitigation measures and monitoring both media output and public opinion. In this regard, web-based tools have emerged as important assets for monitoring epidemiological trends, sharing detailed event schedules and disseminating up-to-date guidance on public health and social measures applied at event venues. For instance, dashboards and mobile applications can enable spectators to assess personal risks during travel, while also allowing health authorities and event organizers to calibrate public health and social measures and risk communication activities accordingly.

Fifth, although Tokyo 2020 and Beijing 2022 were not significant drivers of COVID-19 infections, illness or hospitalizations, their implementation amid ongoing COVID-19 surges did attract attention and stimulate debate, which may have negatively shaped public perceptions of these events. This consideration underscores the importance of addressing public concerns about the negative health consequences of mass gatherings, including the unintended outcomes of risk mitigation measures applied, such as the potential marginalization of specific communities by selective application of such measures, or about unfair treatment for certain types of events. For example, when sporting events are permitted to continue during public health emergencies, but religious congregations are paused. Other equity-related considerations include athletes’ perceptions of fairness in how risk mitigation measures are applied to different sports, or to different competitors within the same sport, such as with respect to time allotted for training and access to training facilities.

Sixth, while safety should always remain a top priority in event planning, organizers must strike a balance between mitigating the health risks associated with holding an event and acknowledging the event’s anticipated health, spiritual, emotional and financial benefits. Applying a risk-based approach can help event planners safely adapt to evolving epidemiological contexts and varying levels of health system capacity, especially during protracted emergencies like COVID-19, and facilitate efforts to strike a balance between risks and benefits. While striking this balance often proves challenging, it can also stimulate innovative approaches to risk assessment and public health practice, as exemplified by these major sporting events.

Seventh, lessons identified from Tokyo 2020 and Beijing 2022 further underscore the contrast between organizing large-scale sporting events and grassroots sports gatherings during the COVID-19 pandemic. Although the economic stakes of modifying, postponing or cancelling large-scale events are much higher, the institutions planning these events are more likely to have the financial means to withstand such measures. Although organizers of grassroots sports events have ingeniously adapted to the constraints posed by COVID-19,^25^ smaller events – which largely rely on ticket sales to generate revenue versus sponsorships or television rights – remain disproportionately affected by limited access to the guaranteed audiences, levels of capital and human resources required to implement certain precautionary measures. Similarly, small business enterprises may be disproportionately affected by industry event cancellations and postponements. Policy-makers, therefore, should ensure that risk mitigation directives do not inadvertently exacerbate baseline societal inequities or create unbalanced burdens for smaller-scale gatherings.

Eighth, while the experiences of Tokyo 2020 and Beijing 2022 have helped generate best practices for mass gathering preparedness, they have also underscored the lack of routine mechanisms for codifying, sharing and institutionalizing lessons identified from mass gatherings held during public health emergencies. Conducting in-depth case studies illustrating how the risk-based approach was applied and calibrated across diverse contexts – as well as providing platforms to share experiences between event organizers, public health experts and other relevant stakeholders – could help expand the evidence base for best practices in mass gathering preparedness.

Finally, metrics to evaluate successes and failures in mass gathering delivery are needed. Qualitative analyses of the perceptions and views of event workers, athletes, spectators and other stakeholders involved in delivering the event in question could inform development of such metrics. Quantitative analyses that examine cases of disease connected to gatherings, identify chains of transmission leading to secondary or tertiary cases, and compare burdens of disease across host jurisdictions could serve as additional metrics of success.

## Conclusion

Mass gatherings should be regarded as integral parts of society, as they provide a broad range of financial, public health and social benefits to host countries, participants and spectators; promote local and global economies; and support legacies of health systems strengthening – through enhanced surveillance and measures for prevention, preparedness and response that remain in place after the event – and knowledge sharing. With appropriate planning and early, proactive engagement of all relevant stakeholders, mass gatherings can be held successfully, including amidst a pandemic, and contribute to increased public awareness of health threats, stronger public health systems, improved partnerships between sectors and conducive environments for safe and healthy participation in public, civic and private life.^3^ Such legacies are especially valuable in settings that host gatherings regularly, and can ensure swift responses to a wide range of complex health risks.^26^ By enforcing mass gathering planning considerations within broader frameworks for public health preparedness, event planners, decision-makers and practitioners can lay the groundwork for safe, successful events amid emerging and evolving public health crises.
